# Anti-Inflammatory Triterpene Glycosides from the Roots of *Ilex dunniana* Levl

**DOI:** 10.3390/molecules22071206

**Published:** 2017-07-19

**Authors:** Yu-Sheng Shi, Yan Zhang, Wen-Zhong Hu, Xi Chen, Xin Fu, Xia Lv, Li-Hong Zhang, Ning Zhang, Guang Li

**Affiliations:** 1 Key Laboratory of Biotechnology and Bioresources Utilization, Educational of Minister, College of Life Science, Dalian Nationalities University, Dalian 116600, China; shiyusheng@dlnu.edu.cn (Y.-S.S.); hwz@dlnu.edu.cn (W.-Z.H.); lvxia@dlnu.edu.cn (X.L.); 2 State Key Laboratory of Bioactive Substance and Function of Natural Medicines, Institute of Materia Medica, Chinese Academy of Medical Sciences and Peking Union Medical College, Beijing 100050, China; 3 Jiamusi College, Heilongjiang University of Chinese Medicine, Jiamusi 154007, China; tonyyan20081982@163.com (Y.Z.); vip12598@163.com (L.-H.Z.); zhangningjms@sina.com (N.Z.); 4 Institute of Medicinal Plant Development, Chinese Academy of Medical Sciences and Peking Union Medical College, Beijing 100193, China; 5 Yunnan Branch, Institute of Medicinal Plant Development, Chinese Academy of Medical Sciences and Peking Union Medical College, Jing Hong 666100, China; liguangshengyao@sina.com; 6 Department of Pharmacognosy, Heilongjiang University of Chinese Medicine, Harbin 150040, China; xiahm_jn@163.com

**Keywords:** triterpene glycosides, *Ilex dunniana* Levl, anti-inflammatory

## Abstract

A new triterpene glycoside ilexdunnoside A (**1**) and a new sulfated triterpene derivative ilexdunnoside B (**2**), together with five known analogues **3**–**7** were isolated from the roots of *Ilex dunniana* Levl. The structures were established by NMR spectroscopic analysis and acid hydrolysis. Results of an in vivo study of the biological activity showed that 75% ethanol and *n*-butanol extracts of the plant displayed anti-inflammatory activities against ear edema in mice, with inhibition rates of 23.5% and 37.5%, respectively, at a dose of 50 mg/kg. Furthermore, Compounds **1**, **2** and **3** exhibited moderate indirect inhibitory effects on lipopolysaccharide-induced NO production in BV2 microglial cells in vitro, with IC_50_ values of 11.60, 12.30 and 9.70 μM, respectively.

## 1. Introduction

**Figure 1:**
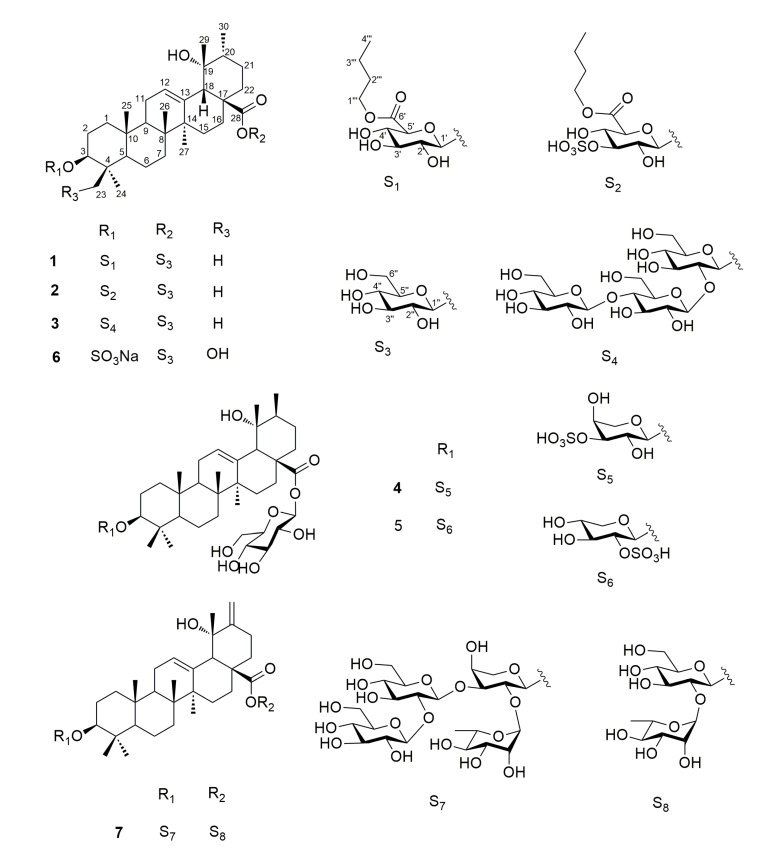
Structures of compounds **1**–**7**.

Inflammation is an important pathophysiological factor of many diseases, such as pneumonia, intestinal catarrh, rheumatoid arthritis, infectious diseases and so on. Plants from the genus *Ilex* (Aquifoliaceae) have been used in the clinic for clearing away inflammation, relieving cough, eliminating phlegm, and so on [1]. In order to search for anti-inflammatory active ingredients, we have performed systematical chemical and pharmacological investigations of *Ilex dunniana* Levl, an evergreen shrub or dungarunga widely distributed to the south of the Qinling mountains and Yangtze Valley in China. Herein we report the isolation and structural elucidation of two new triterpene glycosides, ilexdunnoside A (**1**) and ilexdunnoside B (**2**) (Figure 1), together with five known analogues **3**–**7** from the roots of *Ilex dunniana* Levl, as well as the anti-inflammatory activity of all the isolated compounds.

## 2. Results and Discussion

**Figure 2:**
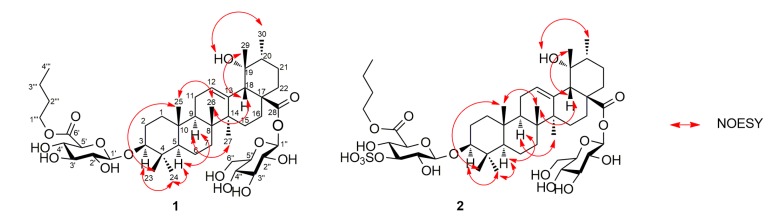
Key NOESY correlations of **1** and **2**.

Ilexdunnoside A (**1**) was isolated as a white, amorphous powder. Its molecular formula was established as C_46_H_74_O_15_ by HRESIMS (*m*/*z* 865.4943 [M − H]^−^, calcd for 865.4955) and NMR data, implying 10 degrees of unsaturation. The ^1^H-NMR spectrum (see Table 1 and Figure S1) showed six tertiary methyl groups at δ_H_ 0.86, 0.96, 1.15, 1.33, 1.39, and 1.69 (each 3H, s), a secondary methyl at δ_H_ 1.06 (3H, d, *J* = 8.0 Hz), an oxymethine proton at δ_H_ 3.32 (1H, dd, *J* = 11.7, 4.5 Hz), and an olefinic proton at δ_H_ 5.53 (1H, br s). The ^13^C-NMR spectrum (see Table 1 and Figure S2) exhibited 46 carbon resonances, of which 30 were attributed to the aglycone. Signals at δ_C_ 89.2 (C-3), 72.5 (C-19), 128.3 (C-12), 139.2 (C-13), and 176.9 (C-28) were assigned to an oxygenated sp^3^ methine, an oxygenated sp^3^ quaternary, a pair of typical olefinic carbons and a carboxy carbon, respectively. The aforementioned data suggested that **1** was a pentacyclic triterpenoid derivative, similar to 3β,19α-dihydroxy-urs-12-en-28-oic acid [2], with the primary difference being the presence of a d-glucuronic acid (GlcA), a d-glucose (Glc), and a *n*-butyl in **1**. The relative configuration of the aglycone of **1** was elucidated based on the spectrum (Figure 2). The correlations of H-3/Me-24, H-5/Me-24, H-5/H-9, H-9/Me-27, OH-19/Me-30 confirmed that these groups were cofacial and α-oriented. Likewise, correlations of Me-23/Me-25, Me-25/Me-26, H-18/Me-26, H-18/Me-29 showed the β-orientation of these groups. The absolute configuration of the aglycone of **1** was presumed to be 3*S*, 5*R*, 8*R*, 9*R*, 10*R*, 14*S*, 17*S*, 18*S*, 19*R*, and 20*R*, by the hypothetical biogenetic pathway.

**Figure 3:**
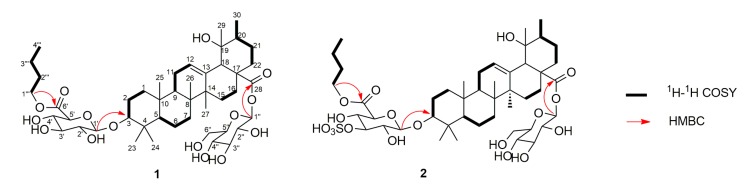
Key ^1^H-^1^H COSY and HMBC correlations of **1** and **2**.

In the ^1^H-NMR spectrum, the sugar portion showed two anomeric proton signals at δ_H_ 4.95 (1H, d, *J* = 7.7 Hz) and δ_H_ 6.26 (1H, d, *J* = 8.0 Hz), with corresponding anomeric carbons at δ_C_ 107.3 and 95.7, respectively. The sugar units were confirmed to be d-glucuronic acid (GlcA) and d-glucose (Glc) by GC analysis of their chiral derivatives after the hydrolysis of **1** with 2 N CF_3_COOH. The ^1^H-^1^H COSY and HSQC spectra revealed the presence of an *n*-butyl in **1**, while HMBC correlations from H_2_-1‴ to C-6′ (170.3) (Figure 3), indicated the attachment of the *n*-butyl to C-6′ Relative large coupling constants of ^3^*J*_H1__’__–H2__’_ (7.7 Hz) and ^3^*J*_H1″__–H2″_ (8.0 Hz) of (6′-BuO) GlcA and Glc in their pyranose form indicated the β-anomeric orientation of them. HMBC correlations from H-1′ to C-3 (δ_C_ 89.2), and from H-1″ to C-28 (δ_C_ 176.9) indicated that the anomeric carbons of (6′-BuO) GlcA and Glc were connected to C-3 and C-28 of the aglycone, respectively. Thus, compound **1** was identified as 3β-(6-*O*-*n*-butyl-d-glucuronopyranosyl)-19α-hydroxy-urs-12-en-28-oic acid 28-*O*-β-d-glucopyranoside, and named ilexdunnoside A.

Ilexdunnoside B (**2**) was obtained as a white, amorphous powder. It had the molecular formula of C_46_H_74_O_18_S according to the deprotonated molecule at *m*/*z* 945.4538 [M − H]^−^ (calcd for 945.4523) in the HRESIMS, suggesting that there was a sulfate group in the molecule. The ^1^H- and ^13^C-NMR spectra showed the characteristic resonances of a 3β,19α-dihydroxy-urs-12-en-28-oic acid skeleton, similar to those of **1**, the only difference being in the sugar portion of **2**. The NMR data showed the presence of an unusual d-(3-*O*-sulphonyl-6-*O*-*n*-butyl)-glucuronopyranosyl unit in **2** instead of the d-(6-*O*-*n*-butyl)-glucuronopyranosyl moiety in **1**, as supported by the downfield shifts of the H-3′ (δ_H_ 5.16) and C-3′ (δ_C_ 83.6) of **2** compared with H-3′ (δ_H_ 4.26) and C-3′ (δ_C_ 77.9) of **1**. Acid hydrolysis of **2**, followed by treatment with BaCl_2_, obtained a white precipitate, also demonstrating the presence of a sulfate group. On the basis of the NOESY spectrum, the relative configuration of **2** was identical to that of **1**. Using the hypothetical biogenetic pathway permitted the assignment of the absolute configuration of **2** as 3*S*, 5*R*, 8*R*, 9*R*, 10*R*, 14*S*, 17*S*, 18*S*, 19*R* and 20*R*. Therefore, the structure of compound **2** was elucidated as 3β-(3-*O*-sulphonyl-6-*O*-*n*-butyl-d-glucuronopyranosyl)-19α-hydroxy-urs-12-en-28-oic acid 28-*O*-β-d-glucopyranoside, and named ilexdunnoside B.

The five known compounds were identified as hylonoside II (**3**), ilexpublesnin H (**4**), ilexpublesnin I (**5**), ilexoside XXXV (**6**), ilekudinoside F (**7**), based on the analysis and comparison of their experimental physical and spectroscopic data with literature values [3,4,5,6]. Compounds **1**–**7** are triterpene glycosides, of which compounds **4** and **5** belong to the sulfated triterpene glycoside class of compounds.

Microglial cells, which are regarded as the most important immune cells in the central nervous system (CNS), are activated by brain injuries. Following activation by bacterial toxins, microglial cells secrete a wide range of inflammatory mediators, such as nitric oxide (NO), tumor necrosis factor-α (TNF-α), interleukin (IL)-1β, and prostanoids [7]. Nitric oxide (NO) plays an important role in the inflammatory process, and an inhibitor of NO production may be considered as a potential anti-inflammatory agent [8]. NO is a physiological messenger that triggers a variety of actions in several systems [9]. It can modulate the release of various inflammatory mediators from a wide range of cells participating in inflammatory responses. Because of its anti-inflammatory properties and cytoprotective effects, adjunctive NO has been considered a plausible means for improving the anti-inflammatory activity. In recent years, NO-releasing drugs have been developed, usually as derivatives of other drugs, which exhibit very powerful anti-inflammatory effects [10,11,12,13].

In order to identify the biological activity, an in vivo anti-inflammatory model were used, the results showed that the 75% ethanol and *n*-butanol extracts of the plant exhibited anti-inflammatory activities against ear edema in vivo, with inhibition rates of 23.5% and 37.5%, respectively, at a dose of 50 mg/kg (as shown in Table 2). Furthermore, as shown in Table 3, in vitro results showed that compounds **1**–**7** obtained from the *n*-butanol extract were tested for their inhibitory effects on lipopolysaccharide-induced NO production in mouse microglial cells. Compounds **1**–**3** displayed moderate indirect anti-inflammatory activity, in which, compound **3** showed cytotoxic activity against microglial cell in vitro (Table 4), so the indirect anti-inflammatory activity of **3** partly may be caused by the cytotoxic activity. The other compounds exhibited weak activities for the inhibition of NO production, and did not show cytotoxic activity.

## 3. Experimental Section

### 3.1. General Information

Optical rotations were obtained on a P2000 automatic digital polarimeter (JASCO, Tokyo, Japan). NMR spectra were measured on a Mercury-400 spectrometer (Varian, Palo Alto, CA, USA). HRESIMS spectra were acquired with an Agilent Technologies 6250 Accurate-Mass Q-TOF LC/MS spectrometer (Agilent, Santa Clara, CA, USA). The MPLC system (Biotage, Uppsala, Sweden) was equipped with an YMC-Pack ODS-A column (500 mm × 50 mm, 50 μm, YMC, Tokyo, Japan). Column chromatography was conducted with MCI GEL CHP20P resin (75–150 μm, Mitsubishi, Tokyo, Japan) and Sephadex LH-20 (Pharmacia Biotech AB, Uppsala, Sweden). TLC was carried out with glass precoated with silica gel GF_254_. BV2 microglial cells were obtained from the Cell Bank of the Chinese Academy of Sciences (Shanghai, China). A microplate reader (Thermo Fisher Scientific, Waltham, MA, USA) was used for the cytotoxicity assays.

### 3.2. Plant Material

The roots of *Ilex dunniana* Levl were collected in Mount Emei, Sichun Province, People’s Republic of China, in August 2015, and were identified by Associate Prof. Xiao-Zhong Chen from Heilongjiang University of Chinese Medicine. A voucher specimen (ID-g-20150828) was deposited at the herbarium of the Jiamusi College, Heilongjiang University of Chinese Medicine.

### 3.3. Extraction and Isolation

The air-dried, powdered root of *Ilex dunniana* Levl (5 kg) were extracted with 75% EtOH (10 L × 1 h × 3). A dried extract (800 g), obtained after concentration in vacuo, was suspended in H_2_O, and partitioned with petroleum ether (60–90 °C), EtOAc, and *n*-BuOH, successively. After the solvent was removed, the *n*-BuOH extracts (60 g) was subjected to MCI GEL CHP20P resin (75–150 μm) eluting with H_2_O, 50% EtOH, and 95% EtOH. The 50% EtOH eluate (20 g) was separated by MPLC (5–100% MeOH-H_2_O, 80 mL/min, 6 h) to obtain 30 fractions (Fr. 1–Fr. 30). Fraction 5 (1.0 g) was chromatographed over Sephadex LH-20 using MeOH, to give compound **7** (12.0 mg). Fraction 6 (2.0 g) was subjected to Sephadex LH-20 eluting with MeOH, to obtain compound **3** (8.0 mg). The separation of fraction 9 (1.0 g) was achieved by Sephadex LH-20 using MeOH as the eluent, to afford **4** (5.0 mg) and **5** (6.3 mg). Fractions 10 (3.5 g) and 15 (1.3 g) were also purified by Sephadex LH-20 eluting with MeOH, to give compounds **1** (7.5 mg), **2** (9.5 mg), and **6** (5.0 mg), respectively.

### 3.4. Product Characterization

*Ilexdunnoside A* (**1**): white, amorphous power; [α]${}_{D}^{25}$ + 38.1 (*c* 0.1, CH_3_OH); ^1^H-NMR (C_5_D_5_N, 400 MHz) and ^13^C-NMR (C_5_D_5_N, 100 MHz) see Table 1; HRESIMS *m*/*z* 865.4943 [M − H]^−^ (calcd. for 865.4955, C_46_H_73_O_15_).

*Ilexdunnoside B* (**2**): white, amorphous power; [α]${}_{D}^{25}$ + 56.1 (*c* 0.2, CH_3_OH); ^1^H-NMR (C_5_D_5_N, 400 MHz) and ^13^C-NMR (C_5_D_5_N, 100 MHz) see Table 1; HRESIMS *m*/*z* 945.4538 [M − H]^−^ (calcd. for 945.4523, C_46_H_73_O_18_S).

### 3.5. Acid Hydrolysis of Saponins

Each saponin (1.0 mg) was refluxed with 2 N aqueous CF_3_COOH (10 mL) at 100 °C for 2 h. The reaction mixture was partitioned between H_2_O (10 mL) and CH_2_Cl_2_ (3 × 4 mL). The CH_2_Cl_2_ layer were washed with H_2_O and evaporated to obtain the aglycone. After evaporation in vacuo and removing the acid by adding MeOH, the aqueous extracts were analyzed by GC. Thus, the absolute configuration of the crude sugar was demonstrated as described in the previous papers [14,15].

### 3.6. Detection of the Sulfate Group

Each compound (2–3 mg) was refluxed with 10% HCl (4 mL) for 4 h. After that the reaction mixture was extracted with Et_2_O. Then, an aliquot of the aqueous layer of each sample was treated with 70% BaCl_2_ to yield a white precipitate (BaSO_4_) [16].

### 3.7. In Vitro Anti-Inflammatory Activity Assays

BV2 microglial cells were maintained in RPMI1640 medium at 37 °C in 5% CO_2_. The cells were placed in 48-well plates and preincubated for 24 h, treated with tested triterpene glycosides dissolved in DMSO at various final concentrations (5.0, 10.0, 20.0, 40.0, 80.0 μM) in triplicate for 1 h, and continuously incubated with LPS (1 μg/mL) for 24 h. Dexamethasone (10^−6^ M) was used as the positive control. From each well, the supernatants (100 μL) were mixed with an equal amount of Griess reagent at room temperature for 20 min. The concentration of NO_2_^−^ was measured for the amount of NO by a microplate reader at 570 nm, using sodium nitrite as the standard to calculate the concentration of the nitrite [17,18].

### 3.8. Cytotoxicity Assays

Cell viabilities were measured using the MTT assay. Briefly, BV2 microglial cells were seeded in 96-well plates at concentrations of 1 × 10^5^ cells per well. After incubation for 2 h, the cells were incubated with compounds for 24 h and then washed with PBS three times. Following the washing step, 200 μL of RPMI 1640 medium containing 0.5 mg/mL MTT were added to each well, the cells were then incubated at 37 °C for another 4 h. Finally, the culture medium was removed, and the formazan crystal was dissolved by adding 150 μL of DMSO. Absorbances at 570 nm were measured using a microplate reader.

### 3.9. In Vivo Anti-Inflammatory Assays

On the basis of the inhibition of croton oil-induced ear edema in mice, the topical anti-inflammatory activity of the test compounds was evaluated. Animal experiments were performed in accordance to the Institutional Guidelines for Animal Care and Use of the Chinese Academy of Medical Sciences and Peking Union Medical College. The protocol was approved by the Committee on the Ethics of Animal Experiments of the Chinese Academy of Medical Sciences and Peking Union Medical College (permit pumber: 002973).The croton oil (*Croton tiglium* L., seed oil) was purchased from Sigma-Aldrich (St. Louis, MO, USA). In ICR male mice (18–20 g, *n* = 10 per group), croton oil (0.4 mg) was mixed with acetone (1 mL) to be applied to the left ear (10 μL each side) topically to induce ear edema. The animals were administered with candidate extracts (50 mg/kg) for 1 h, followed by treatment with croton oil for 4 h. After the application of croton oil, the mice were euthanized using sodium pentobarbital, and ear tissues (8 mm diameter punches) were collected for the measurement of the weight of ear patches (left and right). Based on the weight difference between two plugs (8 mm diameter) of the treated (left) and untreated (right) ears, the edematous response was measured. The percentage reduction in edema in treated mice compared with control mice is calculated for the expression of anti-inflammatory activity. Dexamethasone was used as the positive control [18,19].

## 4. Conclusions

The results of croton-oil ear inflammation in mice showed that 75% ethanol and *n*-butanol extracts of powdered root of *Ilex dunniana* Levl exerted anti-inflammatory activity in vivo against ear edema. Bioactivity-guided isolation of the *n*-butanol extract yielded two new triterpene glycoside **1** and **2** together with five known ones **3**–**7**. Compounds **1**–**7** belong to the triterpene glycosides, of which compounds **4** and **5** belong to sulfated triterpene glycosides. Futhermore, in vitro anti-inflammatory activity showed that compounds **1**, **2** and **3** obtained from the *n*-butanol extract exhibited moderate indirect anti-inflammatory activity on LPS-induced proinflammatory factors production in BV2 microglial cells.

## Figures and Tables

**Table 1:** ^1^H- (400 MHz) and ^13^C-NMR (100 MHz) spectral data of **1** and **2** in C_5_D_5_N (δ in ppm).

Position	Compound 1		Compound 2	
	δ_C_	δ_H_^a^ (*J* in Hz)	δ_C_	δ_H_^a^ (*J* in Hz)
1	38.7	0.84, 1.46	38.2	0.76, 1.37
2	26.9	1.85, 2.12	26.4	1.88, 1.74
3	89.2	3.32 dd (11.7, 4.5)	88.9	3.23 dd (12.0, 4.5)
4	39.4		39.0	
5	55.7	0.80	55.2	0.71
6	18.6	1.44, 1.28	18.1	1.37, 1.21
7	33.4	1.41, 1.56	32.9	1.49, 1.31
8	40.4		40.0	
9	47.6	1.74	47.1	1.65
10	36.8		36.4	
11	23.9	2.00	23.4	1.89
12	128.3	5.53 br s	127.8	5.41 br s
13	139.2		138.7	
14	42.0		41.5	
15	29.1	2.44 m, 1.22	28.7	2.31 m, 1.13
16	26.0	3.09 m, 1.99	25.5	2.95 m, 1.97
17	48.6		48.1	
18	54.3	2.90 s	53.9	2.78 s
19	72.5		72.0	
20	42.0	1.33	41.6	1.25
21	26.6	2.00, 1.84	26.0	1.86, 1.99
22	37.6	2.03, 1.82	37.2	1.70, 1.89
23	28.0	1.33 s	27.5	1.17 s
24	16.8	0.96 s	16.3	0.88 s
25	15.5	0.86 s	15.0	0.72 s
26	17.3	1.15 s	16.8	1.02 s
27	24.5	1.69 s	24.0	1.58 s
28	176.9		176.5	
29	26.9	1.39 s	26.1	1.29 s
30	16.6	1.06 d (8.0)	16.1	0.98 d (6.4)
1′	107.3	4.95 d (7.7)	106.2	4.89 d (7.6)
2′	75.3	4.06	73.4	4.02
3′	77.9	4.26	83.6	5.16
4′	73.0	4.48	72.0	4.40 t (9.1)
5′	77.2	4.57	76.1	4.48 d (9.5)
6′	170.3		169.3	
1′′	95.7	6.26 d (8.0)	95.3	6.13 d (8.0)
2′′	73.9	4.20	71.2	4.10
3′′	78.8	4.28	78.2	4.19
4′′	71.1	4.31	70.6	4.20
5′′	79.1	4.02	78.7	3.91 m
6′′	62.2	4.44, 4.38	61.6	4.32, 4.27
1′′′	64.9	4.26	64.6	4.19
2′′′	30.8	1.56	30.3	1.48
3′′′	19.2	1.30	18.7	1.22
4′′′	13.7	0.75 t (7.3)	13.2	0.68 t (7.1)

^a^ Multiplicity is not clear for some signals due to overlapping.

**Table 2:** Effects of the plant extracts on ear edema induced by croton oil in mice.

Extracts	Dose (mg/kg)	Edema Degree (mg)	Inhibitation Rate (%)
75% Ethanol extract	50.0	15.31 ± 2.01 *	23.5
*n*-Butanol extract	50.0	12.50 ± 1.50 **	37.5
Dexamethasone ^a^	1.0	5.80 ± 0.90 **	71.0
Control group	-	20.01 ± 2.31	-

^a^ Positive control; * *p* < 0.05 vs. control group; ** *p* < 0.01 vs. control group.

**Table 3:** Inhibitory effects of compounds against LPS-induced NO production in mouse BV2 microglial cells (*n* = 3).

Compounds	IC50 (μM)	Compounds	IC50 (μM)
**1**	11.60 ± 0.89	**5**	50.7 ± 3.25
**2**	12.30 ± 1.21	**6**	22.3 ± 2.23
**3**	9.70 ± 0.86	**7**	55.2 ± 3.26
**4**	33.5 ± 2.11	dexamethasone ^a^	0.03

^a^ Positive control.

**Table 4:** The cytotoxic activity of compounds on BV2 microglial cells (*n* = 3).

Compounds	Cell Viability				
	5.0 μM	10.0 μM	20.0 μM	40.0 μM	80.0 μM
**1**	99.74 ± 1.21	99.28 ± 1.32	99.35 ± 1.26	99.62 ± 1.89	99.70 ± 1.36
**2**	98.32 ± 2.05	98.25 ± 1.87	98.16 ± 1.25	98.56 ± 1.37	98.26 ± 1.88
**3**	91.74 ± 1.11 *	90.28 ± 1.82 *	89.35 ± 1.35 *	88.62 ± 1.21 *	88.50 ± 1.32 *
**4**	99.36 ± 1.31	99.37 ± 1.21	99.67 ± 1.32	99.57 ± 1.53	99.33 ± 1.30
**5**	98.21 ± 1.93	99.33 ± 1.54	99.32 ± 1.32	99.21 ± 1.66	99.13 ± 1.28
**6**	102.21 ± 1.03	102.28 ± 1.56	103.35 ± 1.58	103.26 ± 1.28	103.22 ± 1.89
**7**	101.55 ± 1.42	101.68 ± 1.36	101.76 ± 1.47	101.38 ± 1.58	101.68 ± 1.38
Control group	100.00 ± 1.51	100.00 ± 1.26	100.00 ± 1.56	100.00 ± 1.78	100.00 ± 1.55
Dexamethasone ^a^	101.02 ± 1.01	99.98 ± 1.32	100.25 ± 1.67	101.37 ± 1.83	99.78 ± 1.63

^a^ Positive control; * *p* < 0.05 vs. control group.

## Supplementary Materials

The following are available online at http://www.mdpi.com/1420-3049/22/7/1206/s1.
