# Supplementary material for: Anti-Inflammatory Triterpene Glycosides from the Roots of Ilex dunniana Levl
Source: Molecules. 2017 Jul 19;22(7):1206. doi: 10.3390/molecules22071206 (PMC6152231; doi:10.3390/molecules22071206)
Supplement: Supplementary file 1 [file molecules-22-01206-s001.pdf]

Anti-inflammatory Triterpene Glycosides from the Radix of *Ilex dunniana Levl*

Yu-Sheng Shi[1,2,†], Yan Zhang[3,†], Wen-Zhong Hu[1], Xi Chen[4,5,*], Xin Fu[6], Xia Lv[1], Li-Hong Zhang[3], Ning Zhang[3], and Guang Li[5]

Supplementary Materials

List of Contents

Fig. S1 The ^{1}H NMR Spectrum of **1** in C_5D_5N (400 MHz)

Fig. S2 The ^{13}C NMR Spectrum of **1** in C_5D_5N (100MHz)

Fig. S3 The DEPT Spectrum of **1** in C_5D_5N (100MHz)

Fig. S4 The gHSQC Spectrum of **1** in C_5D_5N (400 MHz)

Fig. S5 The gHMBC Spectrum of **1** in C_5D_5N (400 MHz)

Fig. S6 The gCOSYS pectrum of **1** in C_5D_5N (400 MHz)

Fig. S7 The NOESY pectrum of **1** in C_5D_5N (400 MHz)

Fig. S8 HRESIMS spectrum of **1**

Fig. S9 The ^{1}H NMR Spectrum of **2** in C_5D_5N (400 MHz)

Fig. S10 The ^{13}C NMR Spectrum of **2** in C_5D_5N (100MHz)

Fig. S11 The DEPT Spectrum of **2** in C_5D_5N (100MHz)

Fig. S12 The gHSQC Spectrum of **2** in C_5D_5N (400 MHz)

Fig. S13 The gHMBCSpectrum of **2** in C_5D_5N (400 MHz)

Fig. S14 The gCOSYSpectrum of **2** in C_5D_5N (400 MHz)

Fig. S15 The NOESY pectrum of **2** in C_5D_5N (400 MHz)

Fig. S16 HRESIMS spectrum of **2**

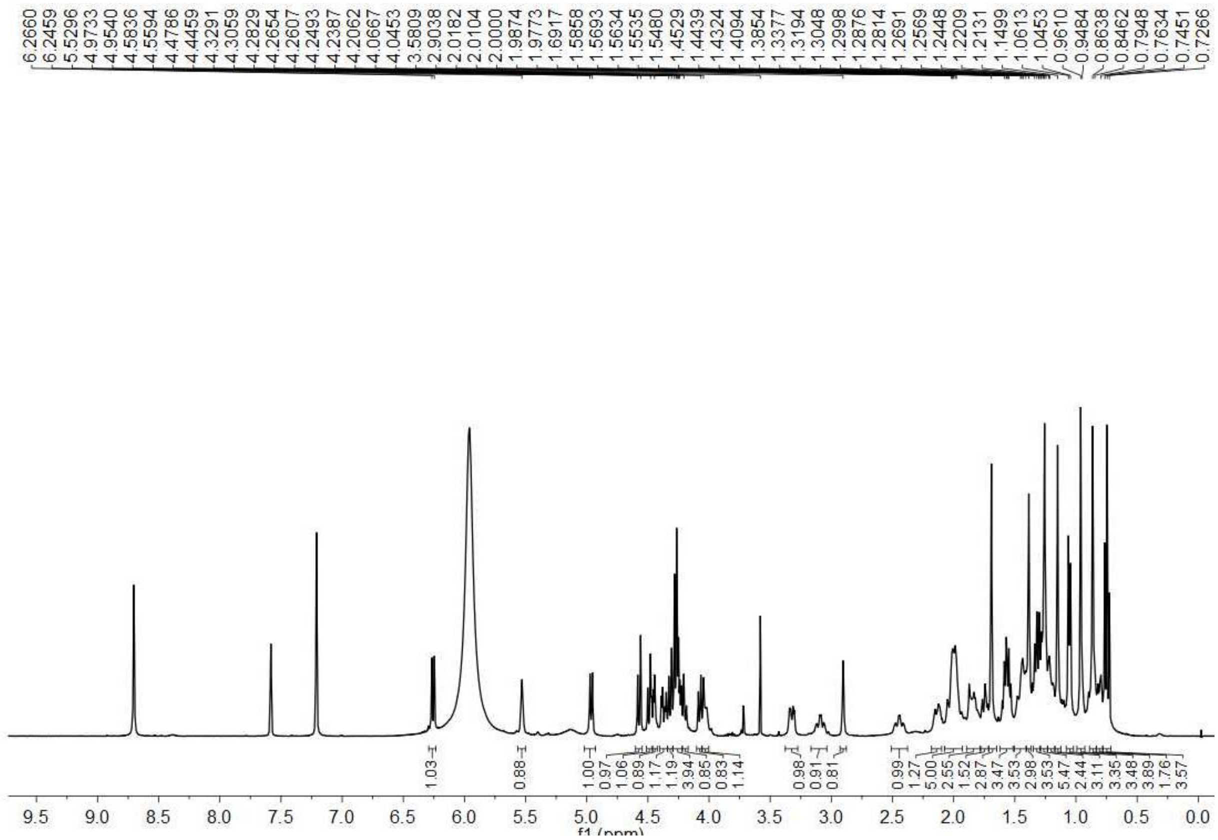

Fig. S1 ^{1}H-NMR spectrum of compound **1** in C_5D_5N (400 MHz)

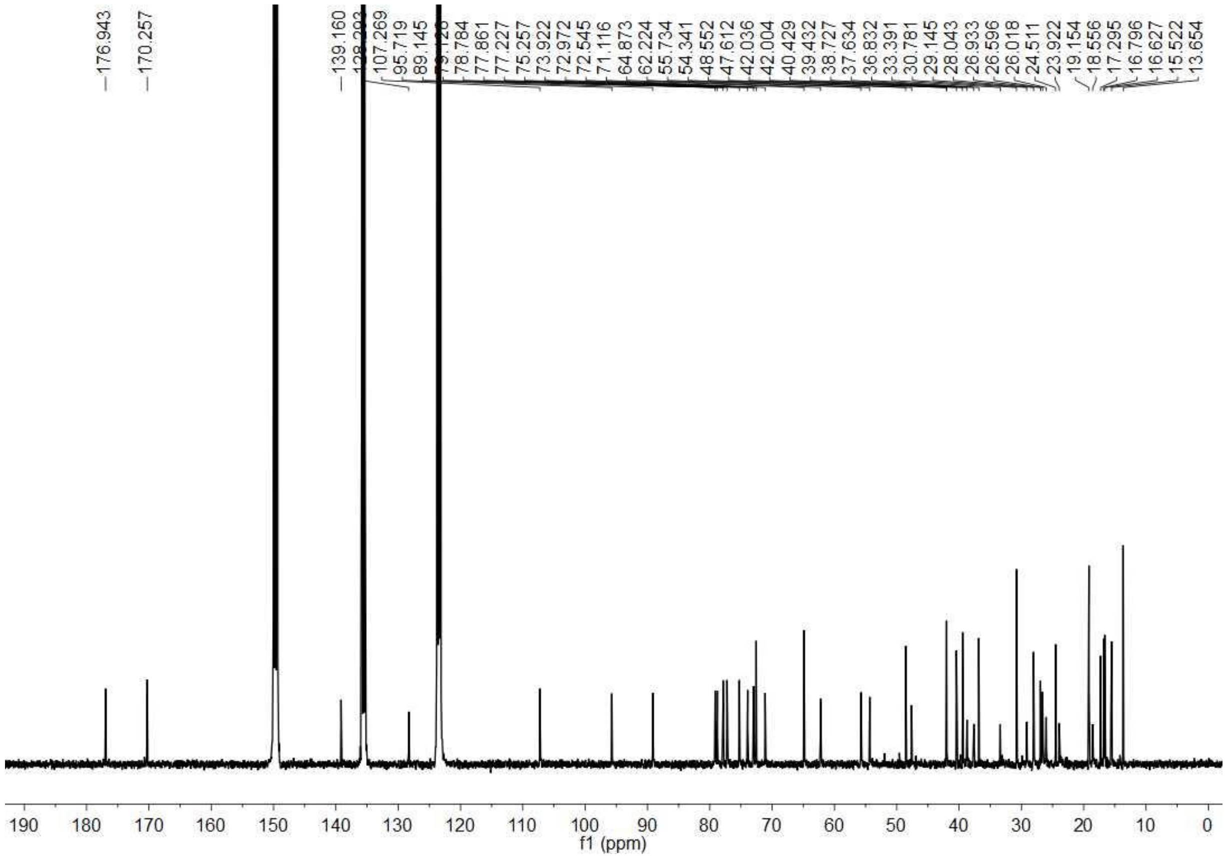

Fig. S2 ^{13}C-NMR spectrum of compound **1** in C_5D_5N (100 MHz)

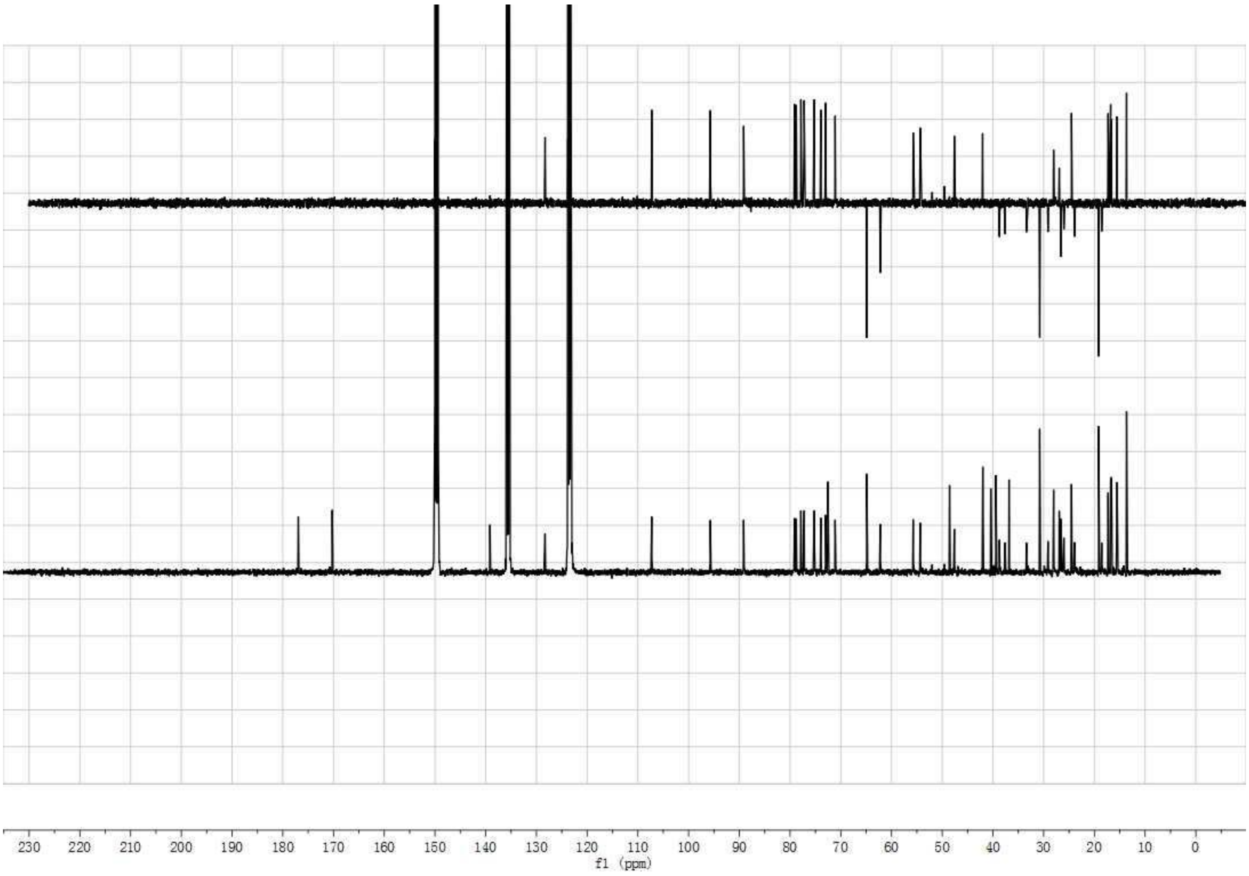

Fig. S3 DEPT spectrum of compound **1** in C_5D_5N (100 MHz)

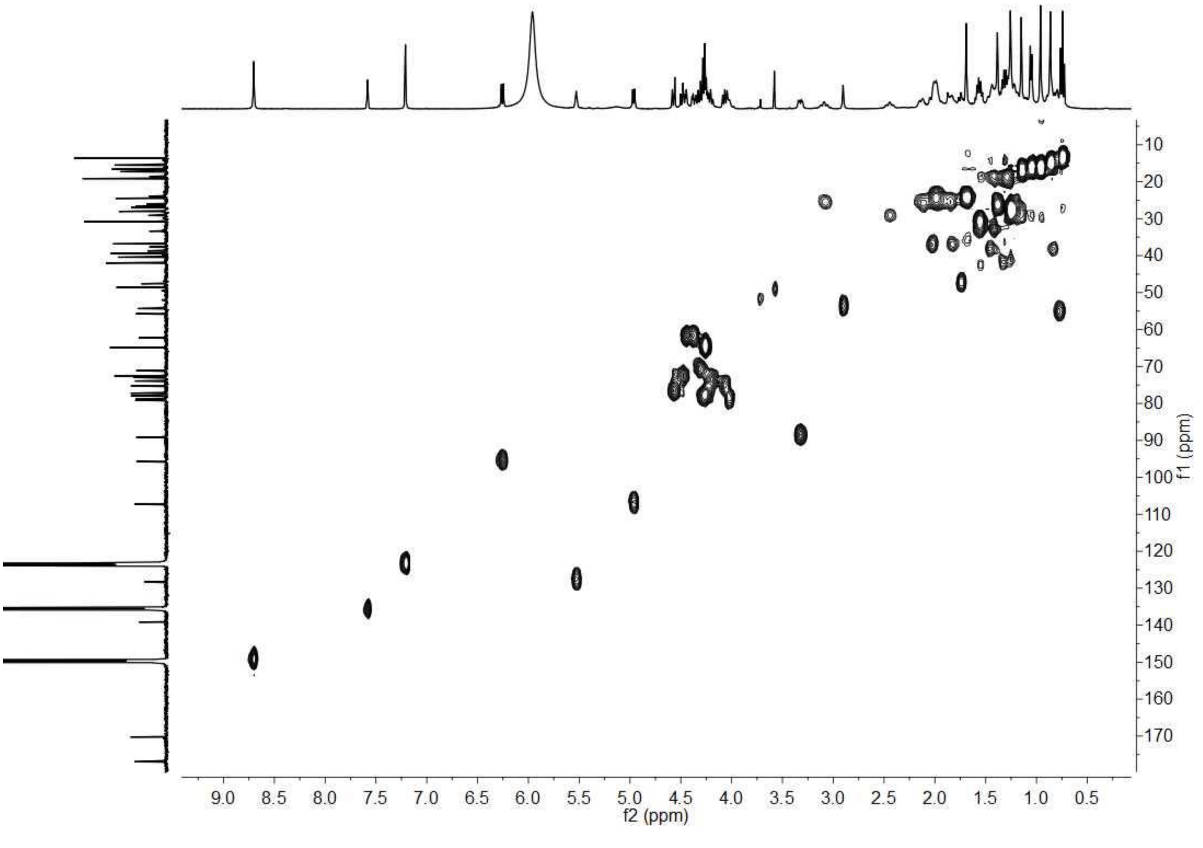

Fig. S4 HSQC spectrum of compound **1** in C_5D_5N (400 MHz)

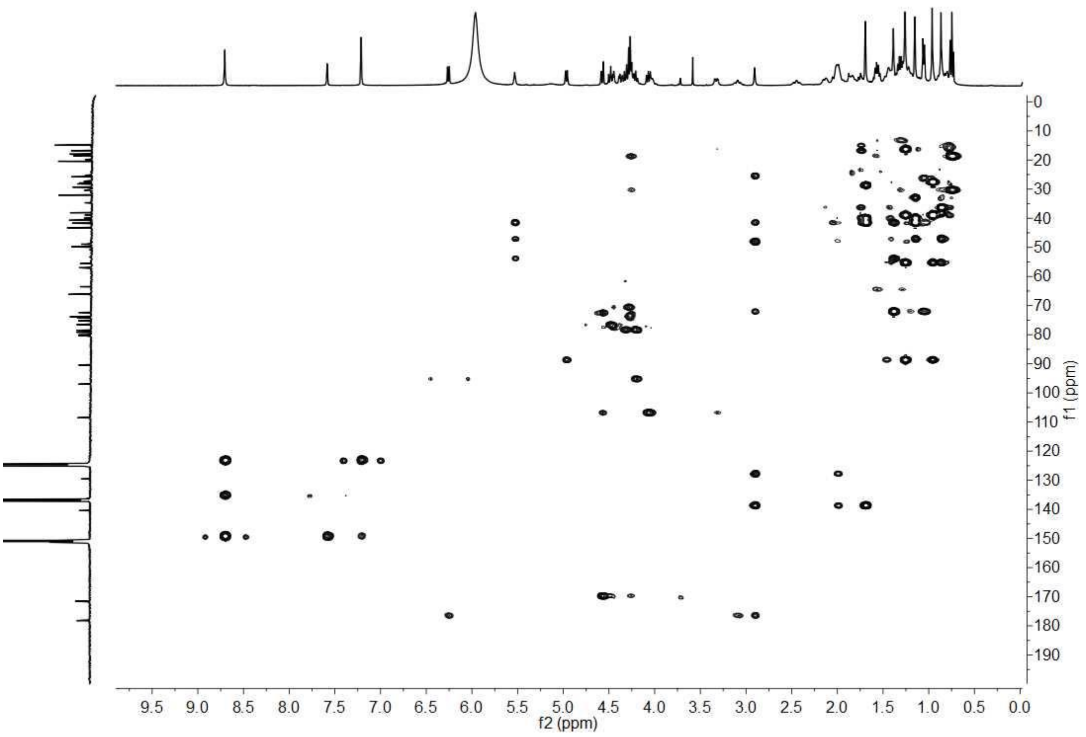

Fig. S5 HMBC spectrum of compound **1** in C_5D_5N (400 MHz)

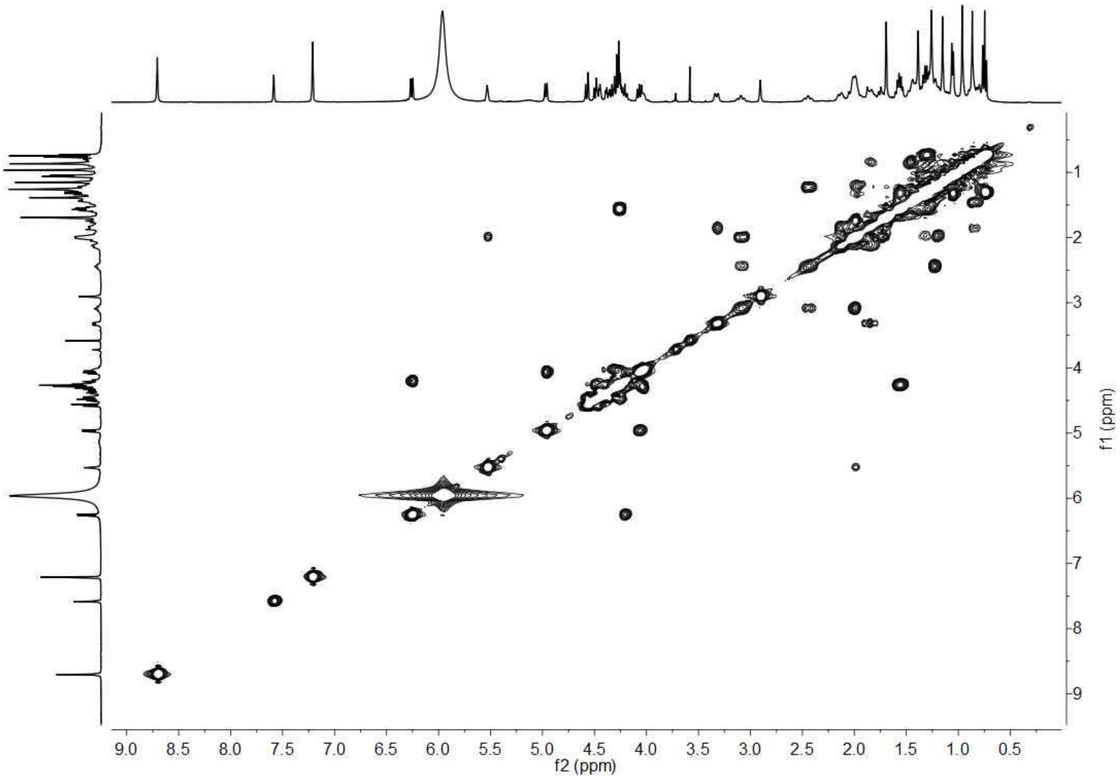

Fig. S6 $^1H-^1H$ COSY spectrum of compound **1** in C_5D_5N (400 MHz)

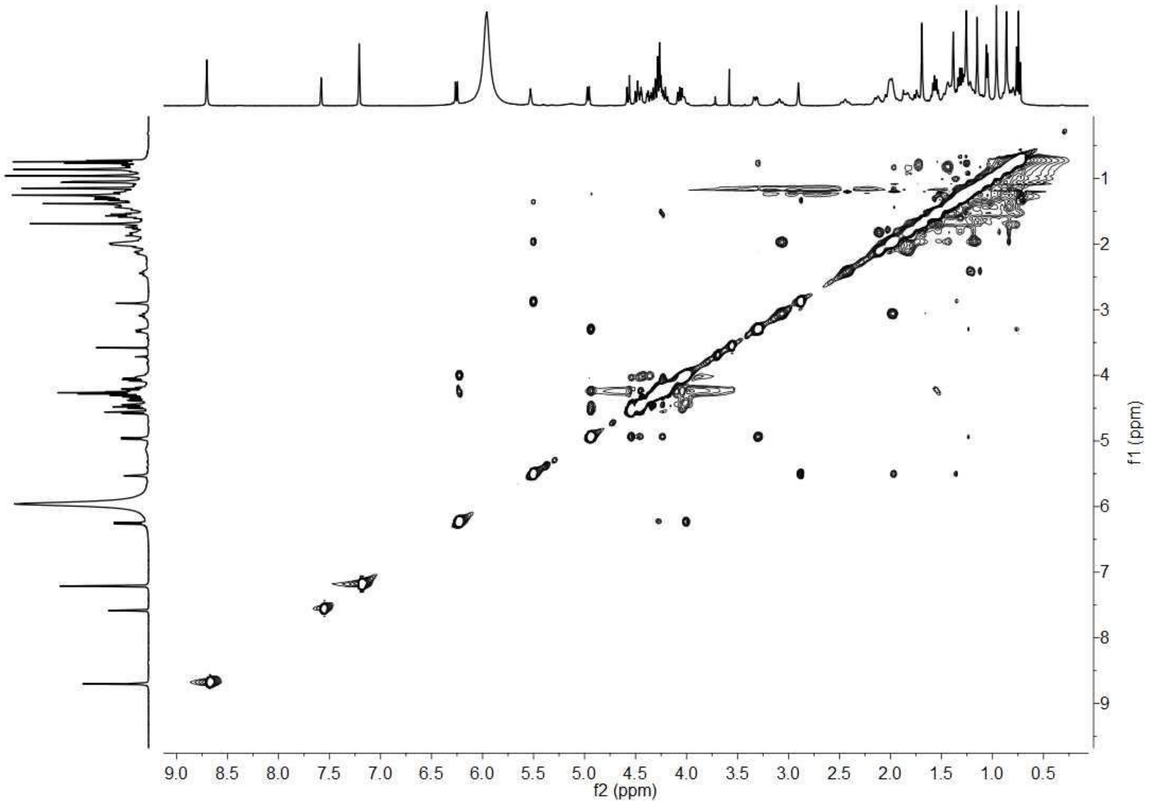

Fig. S7 NOESY spectrum of compound **1** in C_5D_5N (400 MHz)

m/z	Ion	Formula	Abundance
865.4943	(M-H)-	C46 H73 O15	6971.4

Best	Formula (M)	Ion Formula	Calc m/z	Score	Cross Score	Mass	Calc Mass	Diff (ppm)	Abs Diff (ppm)
✓	C46 H74 O15	C46 H73 O15	865.4955	74.12		866.5013	866.5028	1.69	1.6[illegible]

Fig. S8 HRESIMS spectrum of compound **1**

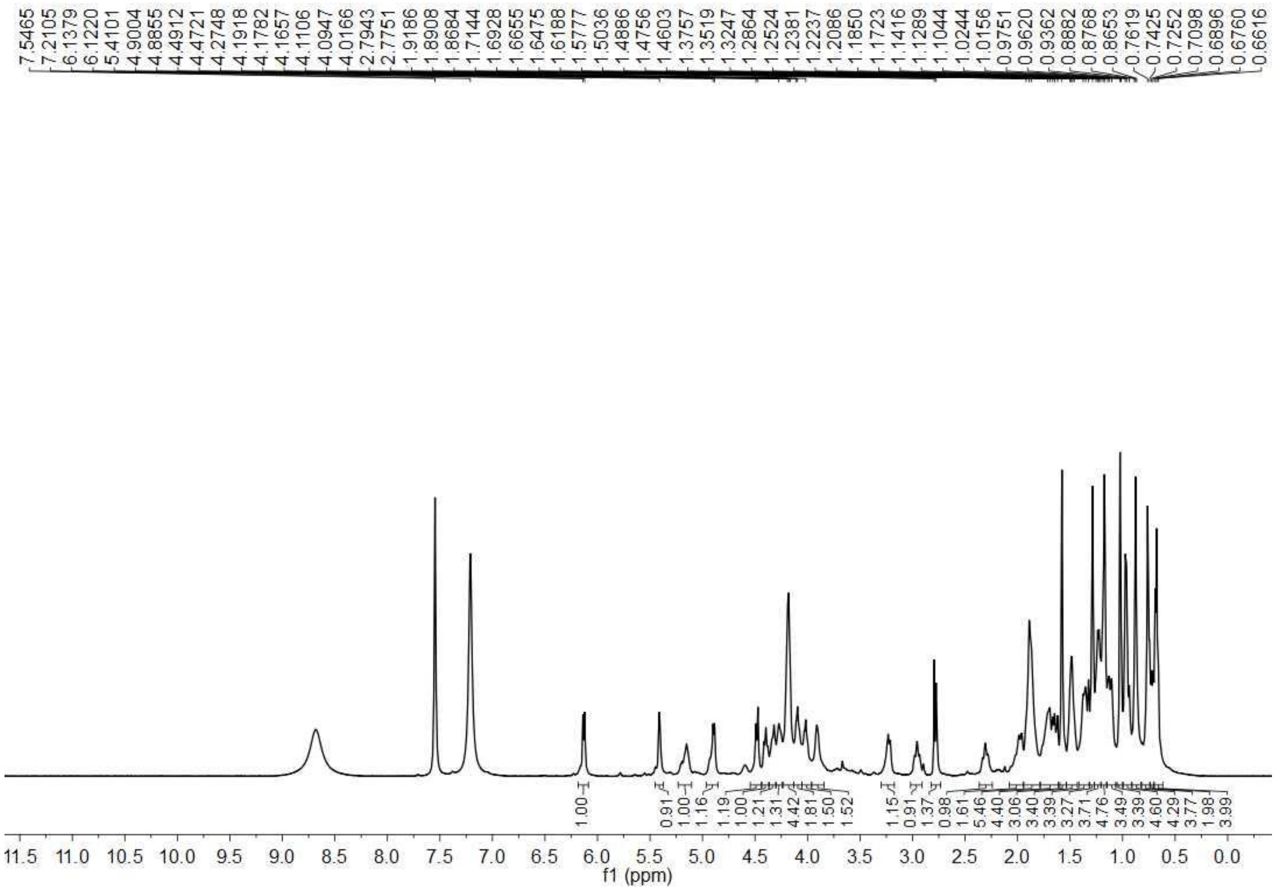

Fig. S9 ^{1}H-NMR spectrum of compound **2** in C_5D_5N (400 MHz)

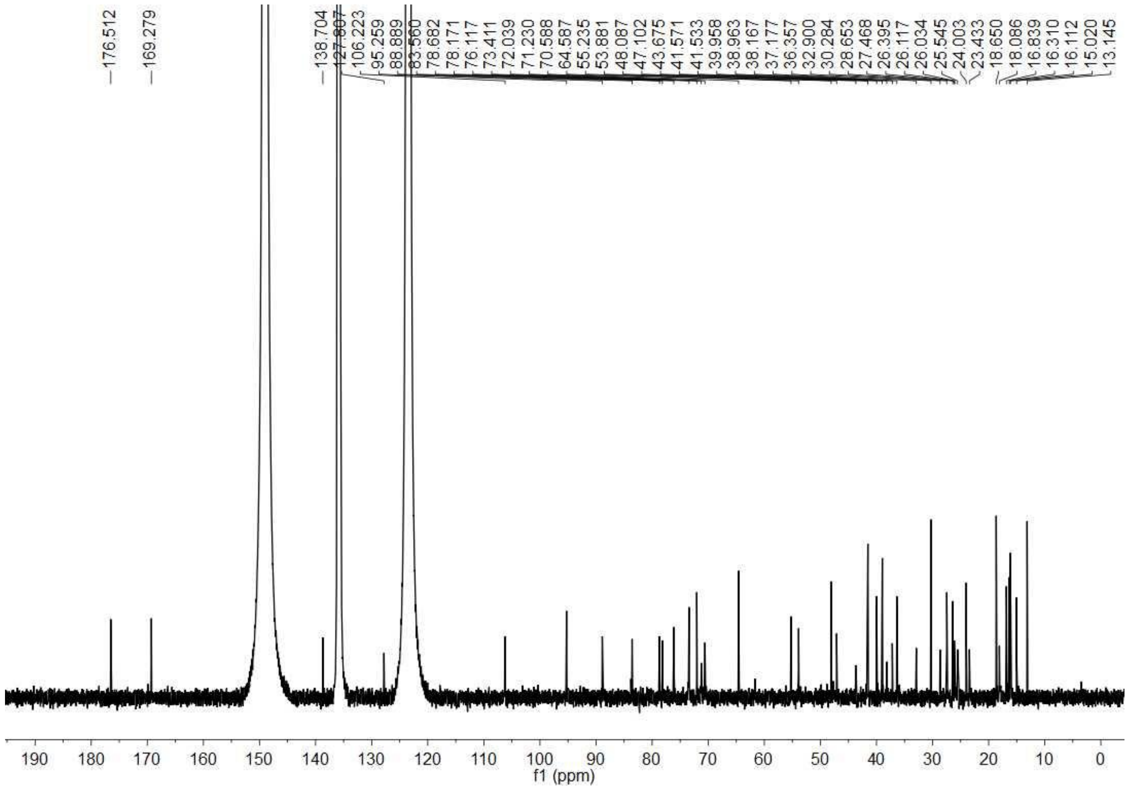

Fig. S10 ^{13}C-NMR spectrum of compound **2** in C_5D_5N (100 MHz)

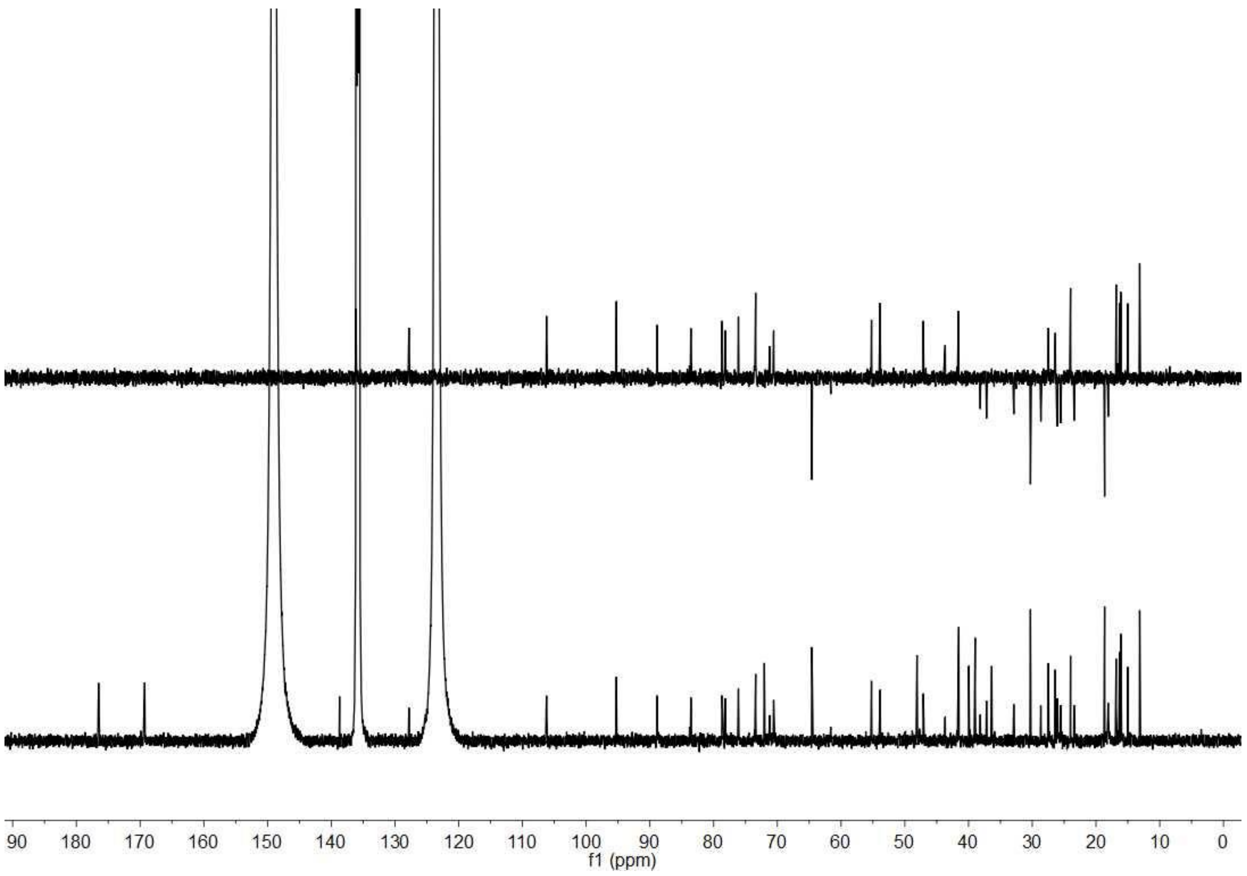

Fig. S11 DEPT spectrum of compound **2** in C_5D_5N (100 MHz)

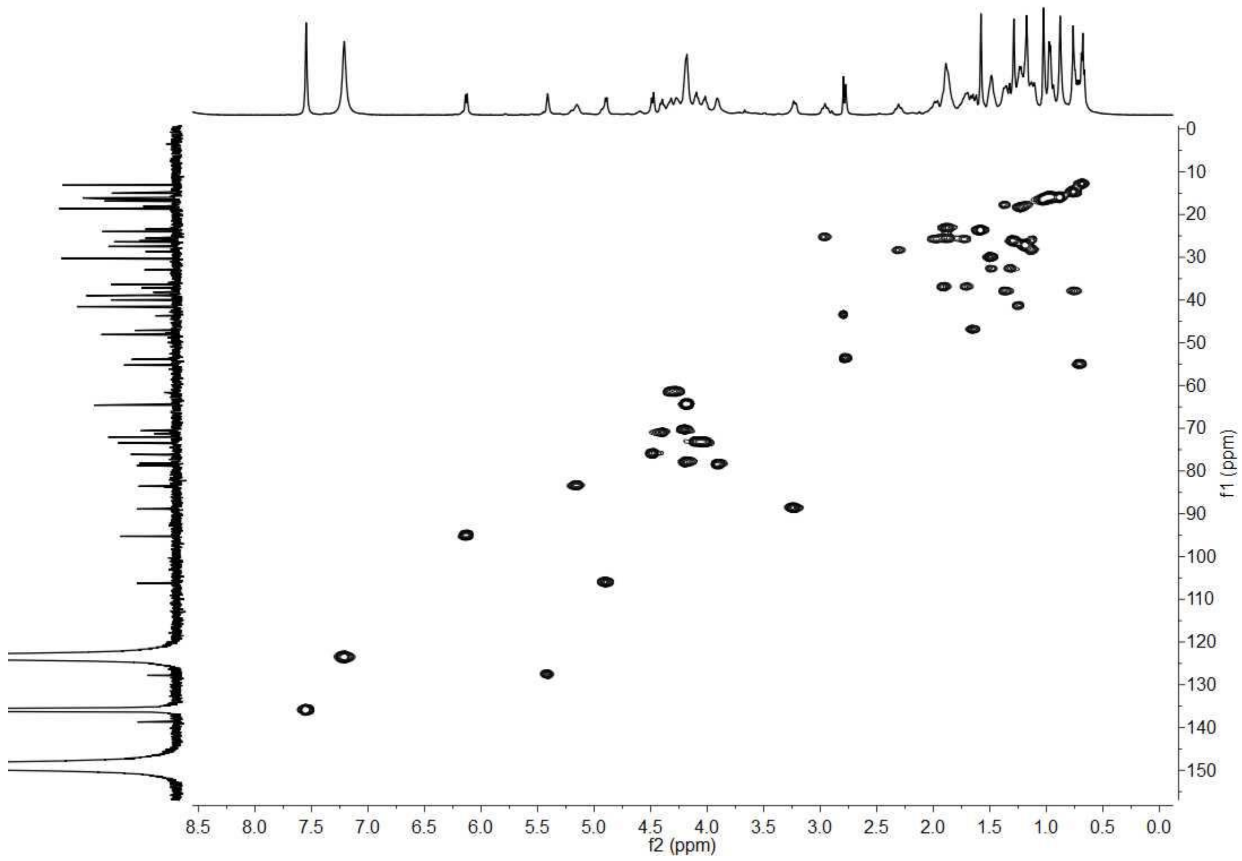

Fig. S12 HSQC spectrum of compound **2** in C_5D_5N (400 MHz)

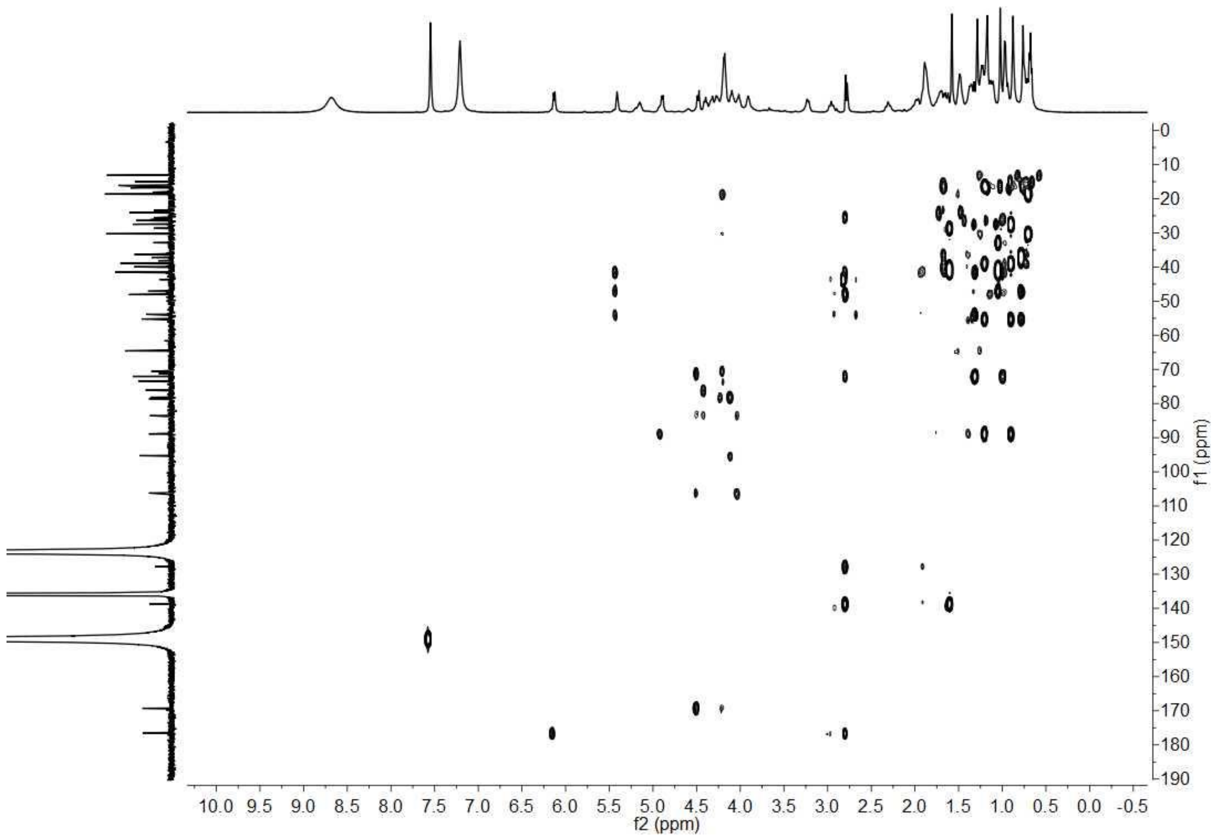

Fig. S13 HMBC spectrum of compound **2** in C_5D_5N (400 MHz)

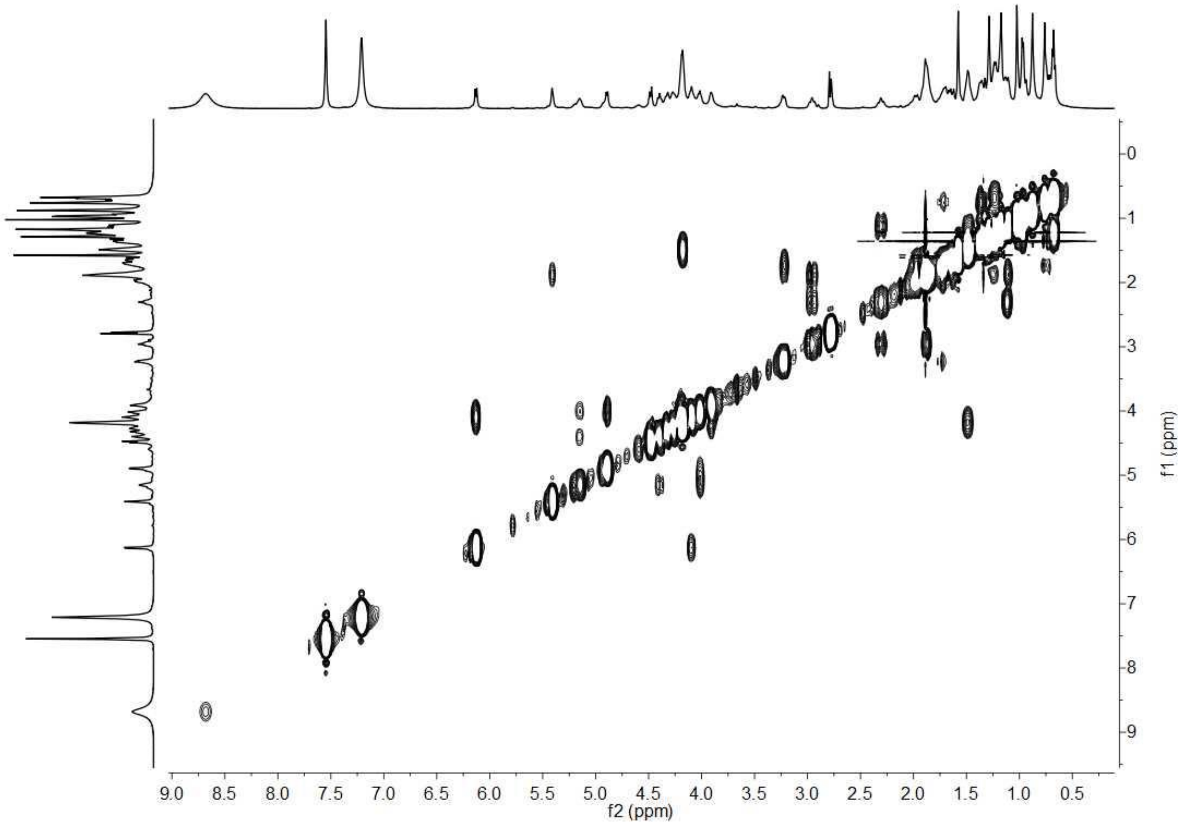

Fig. S14 $^1H-^1H$ COSY spectrum of compound **2** in C_5D_5N (400 MHz)

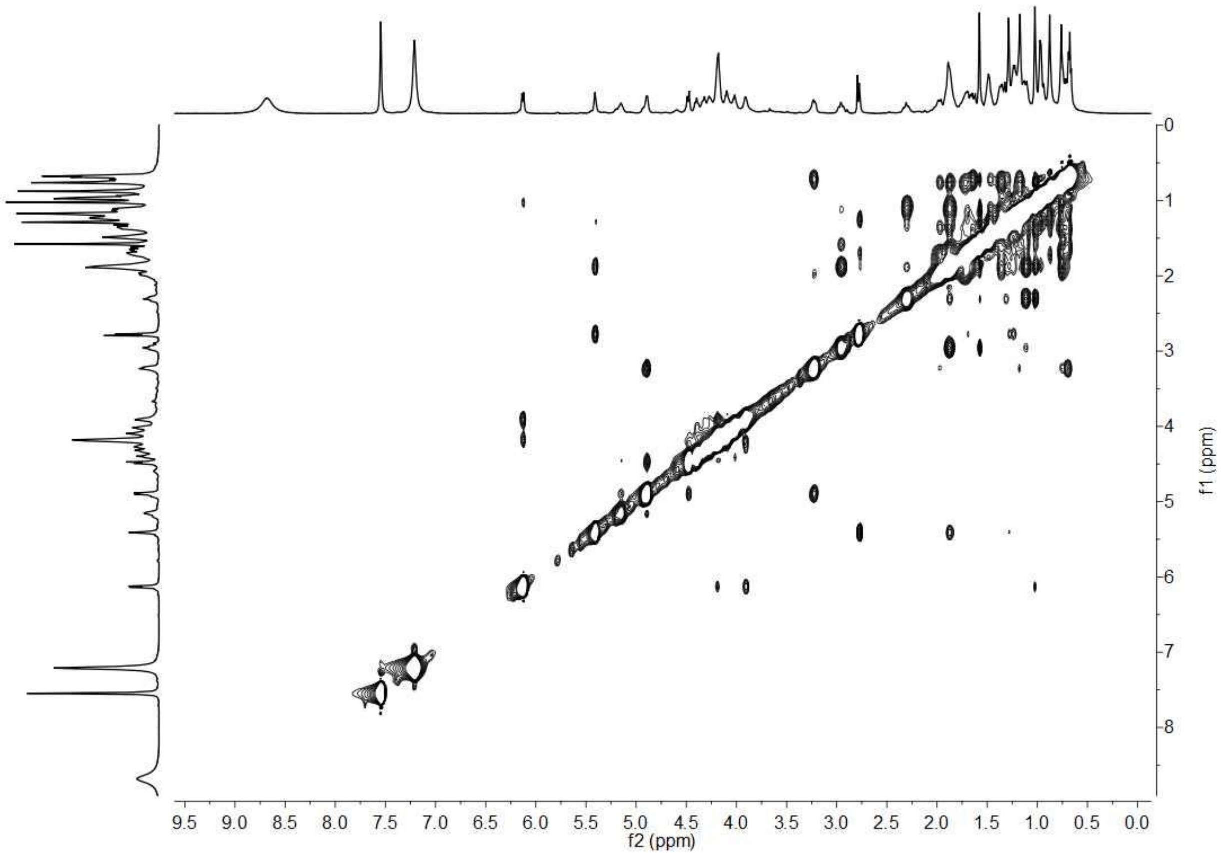

Fig. S15 NOESY spectrum of compound **2** in C_5D_5N (400 MHz)

m/z	Ion	Formula	Abundance
945.4538	(M-H)-	C46 H73 O18 S	32487.1

Best	Formula (M)	Ion Formula	Calc m/z	Score	Cross Score	Mass	Calc Mass	Diff (ppm)	Abs Dif
✓	C46 H74 O18 S	C46 H73 O18 S	945.4523	96.1		946.4612	946.4596	-1.73	

Fig. S16 HRESIMS spectrum of compound **2**
